# Retraction: In silico screening of herbal and nanoparticle lead compounds for effectivity against H5N1, H1N1 neuraminidase and telomerase

**DOI:** 10.1186/1471-2105-11-258

**Published:** 2010-05-18

**Authors:** Sayak Ganguli, Manjita Mazumder, Protip Basu, Paushali Roy, Sayani Mitra, Abhijit Datta

**Affiliations:** 1 DBT-Centre for Bioinformatics, Presidency College, Kolkata, India

## Retraction

This article has been regretfully retracted due to significant plagiarism by the authors. The authors apologise to all affected parties for the inconvenience.
